# Parasite reduction ratio one day after initiation of artemisinin-based combination therapies and its relationship with parasite clearance time in acutely malarious children

**DOI:** 10.1186/s40249-018-0503-7

**Published:** 2018-12-07

**Authors:** Kazeem Akano, Godwin Ntadom, Chimere Agomo, Christian T. Happi, Onikepe A. Folarin, Grace O. Gbotosho, Olugbenga Mokuolu, Finomo Finomo, Joy C. Ebenebe, Nma Jiya, Jose Ambe, Robinson Wammanda, George Emechebe, Oluwabunmi K. Basorun, Olubunmi A. Wewe, Sikiru Amoo, Nnenna Ezeigwe, Stephen Oguche, Bayo Fatunmbi, Akintunde Sowunmi

**Affiliations:** 1 Department of Pharmacology and Therapeutics, University of Ibadan, Ibadan, Nigeria; 2 Antimalarial Therapeutic Efficacy Monitoring Group, National Malaria Elimination Programme, The Federal Ministry of Health, Abuja, Nigeria; 3 Department of Medical Laboratory Science, University of Lagos, Lagos, Nigeria; 4 Department of Biological Sciences and African Centre of Excellence for Genomics of Infectious Diseases (ACEGID), Redeemer University, Ede, Nigeria; 5 Institute for Medical Research and Training, University of Ibadan, Ibadan, Nigeria; 6 Department of Pharmacology and Toxicology, Faculty of Pharmacy, University of Ibadan, Ibadan, Nigeria; 7 Department of Paediatrics, University of Ilorin, Ilorin, Nigeria; 8 Department of Paediatrics, Federal Medical Centre, Yenagoa, Nigeria; 9 Department of Paediatrics, Nnamdi Azikiwe University, Awka, Nigeria; 10 Department of Paediatrics, Uthman Dan Fodio University, Sokoto, Nigeria; 11 Department of Paediatrics, University of Maiduguri, Maiduguri, Nigeria; 12 Department of Paediatrics, Ahmadu Bello University, Zaria, Nigeria; 13 Department of Paediatrics, Imo State University Teaching Hospital, Orlu, Nigeria; 14 Department of Paediatrics, University of Jos, Jos, Nigeria; 15 World Health Organization, Country Office, Kampala, Uganda

**Keywords:** Falciparum malaria, Artemisinin-based combination therapies, Parasite clearance, Children, Nigeria

## Abstract

**Background:**

In acute falciparum malaria, asexual parasite reduction ratio two days post-treatment initiation (PRRD2) ≥ 10 000 per cycle has been used as a measure of the rapid clearance of parasitaemia and efficacy of artemisinin derivatives. However, there is little evaluation of alternative measures; for example, parasite reduction ratio one day after treatment initiation (PRRD1) and its relationship with parasite clearance time (PCT) or PRRD2. This study evaluated the use of PRRD1 as a measure of responsiveness to antimalarial drugs.

**Methods:**

In acutely malarious children treated with artesunate-amodiaquine (AA), artemether-lumefantrine (AL) or dihydroartemisinin-piperaquine (DHP), the relationships between PRRD1 or PRRD2 and PCT, and between PRRD1 and PRRD2 were evaluated using linear regression. Agreement between estimates of PCT using PRRD1 and PRRD2 linear regression equations was evaluated using the Bland-Altman analysis. Predictors of PRRD1 > 5000 per half cycle and PRRD2 ≥ 10 000 per cycle were evaluated using stepwise multiple logistic regression models. Using the linear regression equation of the relationship between PRRD1 and PCT previously generated in half of the DHP-treated children during the early study phase, PCT estimates were compared in a prospective blinded manner with PCTs determined by microscopy during the later study phase in the remaining half.

**Results:**

In 919 malarious children, PRRD1 was significantly higher in DHP- and AA-treated compared with AL-treated children (*P* <  0.0001). PRRD1 or PRRD2 values correlated significantly negatively with PCT values (*P* <  0.0001 for each) and significantly positively with each other (*P* <  0.0001). PCT estimates from linear regression equations for PRRD1 and PRRD2 showed insignificant bias on the Bland-Altman plot (*P* = 0.7) indicating the estimates can be used interchangeably. At presentation, age > 15 months, parasitaemia > 10 000/μl and DHP treatment independently predicted PRRD1 > 5000 per half cycle, while age > 30 months, haematocrit ≥31%, body temperature > 37.4 °C, parasitaemia > 100 000/μl, PRRD1 value > 1000 and no gametocytaemia independently predicted PRRD2 ≥ 10 000 per cycle. Using the linear regression equation generated during the early phase in 166 DHP-treated children, PCT estimates and PCTs determined by microscopy in the 155 children in the later phase were similar in the same patients.

**Conclusions:**

PRRD1 and estimates of PCT using PRRD1 linear regression equation of PRRD1 and PCT can be used in therapeutic efficacy studies.

**Trial registration:**

Pan African Clinical Trial Registration PACTR201709002064150, 1 March 2017, http://www.pactr.org

**Electronic supplementary material:**

The online version of this article (10.1186/s40249-018-0503-7) contains supplementary material, which is available to authorized users.

## Multilingual abstracts

Please see Additional file 1 for translations of the abstract into the five official working languages of the United Nations.

## Background

The rapid clearance of asexual parasitaemia following artemisinin-based combination therapies (ACTs), measured as parasite reduction ratio two days post-treatment initiation (PRRD2) ≥ 10 000 per cycle, is primarily dependent on the artemisinin components and is the hallmark of artemisinin derivatives in sensitive *Plasmodium falciparum* infections [1–3]. The use of PRRD2 is based on the *P. falciparum* asexual intraerythrocytic development cycle of approximately 48 h from a very young ring stage to schizonts, which is equally shared between the appearance of the parasite within the parasitized erythrocytes and sequestration in deep tissue [1, 2].

Synchronization of parasite growth in vitro is a necessary step in evaluating the stage-specific action of antimalarial drugs [4]. However, given most human infections with *P. falciparum* are relatively synchronized and artemisinins have broad stage specificity [5, 6], alternative indices for evaluating rapid or delayed clearance of asexual parasitaemia within a day of commencing ACTs are needed for assessment in therapeutic efficacy studies. One such index with potential clinical application is asexual parasite reduction ratio one day after treatment initiation (PRRD1). The use of PRRD1 is plausible because parasite prevalence one day after initiation of ACTs is also directly attributable to rapid clearance of asexual parasitaemia by artemisinin components of ACTs [2, 5, 6].

An estimated 58 million-plus cases of acute falciparum infections are reported annually in Nigeria [7]. Despite most of the estimated cases occurring in children, there is no reported prospective evaluation of the relationship between PRRD1 or PRRD2 and parasite clearance time (PCT), or of the relationship between PRRD1 and PRRD2 in malarious Nigerian children. Such an evaluation may assist in assessing the rapid clearance of asexual parasitaemia following initiation of treatments and in predicting children most likely to have slow clearance of parasitaemia measured as asexual parasite positivity three days after treatment initiation (APPD3).

This study involved a cohort of acutely malarious children enrolled in a multi-site therapeutic efficacy study of artesunate-amodiaquine (AA), artemether-lumefantrine (AL) or dihydroartemisinin-piperaquine (DHP). The aims were to: i) establish, using simple mathematical approaches, the relationship between PRRD1 and PCT, and PRRD2 and PCT; ii) evaluate the agreement between estimates of PCT derived from PRRD1 and PRRD2 simple mathematical approaches; iii) explore if PRRD1 can be used as a measure of responsiveness of falciparum malaria to ACTs; and iv) determine the factors contributing to PRRD1 > 5000 per half cycle and PRRD2 ≥ 10 000 per cycle. An additional objective was to use the linear regression equation generated from the relationship between PRRD1 and PCT in half of the group of DHP-treated children in the early phase of the ongoing study to estimate PCT in the later phase of the same study in the remaining half, and to compare the estimated PCT in a prospective blinded manner with the PCT determined by microscopy in the same patient.

## Methods

### Study sites and study population

The study took place between June 2014 and December 2015. It was nested in Nigeria’s National Malaria Elimination Programme to monitor the therapeutic efficacy of three ACTs at eight sentinel sites located in six geographical areas of Nigeria, namely: Ogbia, Bayelsa; Neni, Anambra; Ogwa, Imo; Numan, Adamawa; Ilorin, Kwara; Kura, Kano; Bodinga, Sokoto and Ibadan, Oyo (PACTR201709002064150). At these sites, 86, 55, 168, 177, 122, 169, 165 and 50 children aged under five years, respectively, were enrolled in the study.

At virtually all of the sentinel sites, malaria transmission occurs all year round, but it is more intense during the rainy season from April to October. The details of the therapeutic efficacy study from which the present dataset is derived have been reported elsewhere [8].

### Study procedures

Standardized procedures and protocols were used at all sites [8]. Briefly, patients were eligible to participate in the study if: they were aged 6–59 months, had symptoms compatible with acute uncomplicated malaria such as fever, anorexia, vomiting or abdominal discomfort with or without diarrhoea, with *P. falciparum* mono-infections between 2000 and 200 000/μl of blood, a body (axillary) temperature > 37.4 °C or history of fever in the 24–48 h preceding presentation, absence of other concomitant illness, no history of antimalarial drug ingestion in the two weeks prior to enrolment, no evidence of severe malaria [9, 10], and parents or guardians giving written informed consent.

Enrolled patients were randomized to AA, AL or DHP treatments for three days (day 0–2), as previously described [11]. The day of presentation (day of starting treatment) was regarded as day 0. Thick and thin blood films, taken from a finger prick, were obtained from each child as soon as they came to the clinic and the slides were carefully labelled with the patients’ codes and air dried before being Giemsa stained. All drugs were given orally. For children who were not able to swallow whole tablets, the tablets were carefully crushed using a tablet crusher, dissolved in water and administered orally. In patients treated with AA or DHP, the drug was given as single daily doses in the clinic by the physician. In patients treated with AL, the 0, 8, 24 and 48 h doses were given in the clinic by the physician, and the 36 and 60 h doses were given by parents or guardians of the children at home. A phone call was made to remind parents/guardians of the time of the second daily doses of AL and to monitor the outcome of drug administration. Parents or guardians were questioned at follow-up on the time and events after drug administration. After drug administration at the clinic, all patients waited for at least 30 min to ensure the drug was not vomited. If it was, the dose was repeated. If the repeat dose was vomited, the patient was excluded from the study. Follow-up with clinical, parasitological and haematocrit evaluation was done daily on days 1–3 and 7, and thereafter weekly for an additional five weeks. Thick and thin blood films prepared from a finger prick were stained with Giemsa and examined by light microscopy under an oil-immersion objective at 1000 × magnification by two independent assessors who did not know the drug regimen of the patients. A senior member of the study team reviewed the slides if there was any disagreement between the two microscopists. In addition, the slides of every fourth child enrolled in the study were reviewed by a senior member. Asexual parasitaemia in thick films were estimated by counting asexual parasites relative to 500 leukocytes or 500 asexual forms, whichever occurred first. From this figure, the parasite density was calculated assuming a leukocyte count of 6000/μl of blood [12]. Sexual parasites were not quantified but their presence in blood films was noted. A slide was considered parasite negative if no asexual or sexual parasite was detected after examination of 200 microscope fields. Asexual parasite reduction ratio (PRR) one or two days after treatment initiation (PRRD1 or PRRD2) was defined as the ratio of parasitaemia at enrolment and that on day one or two, respectively. Asexual parasite positivity on day 3 (APPD3) was defined as the proportion of children with residual asexual parasitaemia three days after treatment initiation. Parasite clearance time (PCT) was defined as the time elapsing between drug administration and absence of microscopic detection of peripheral asexual parasitaemia [3, 13].

Polymerase chain reaction (PCR) parasite genotyping before and three days after treatment initiation was done using MSP 1 or MSP 2 or both genes, as previously described [14], and compared side by side to detect actual delay in asexual parasite clearance.

**Fig. 1:**
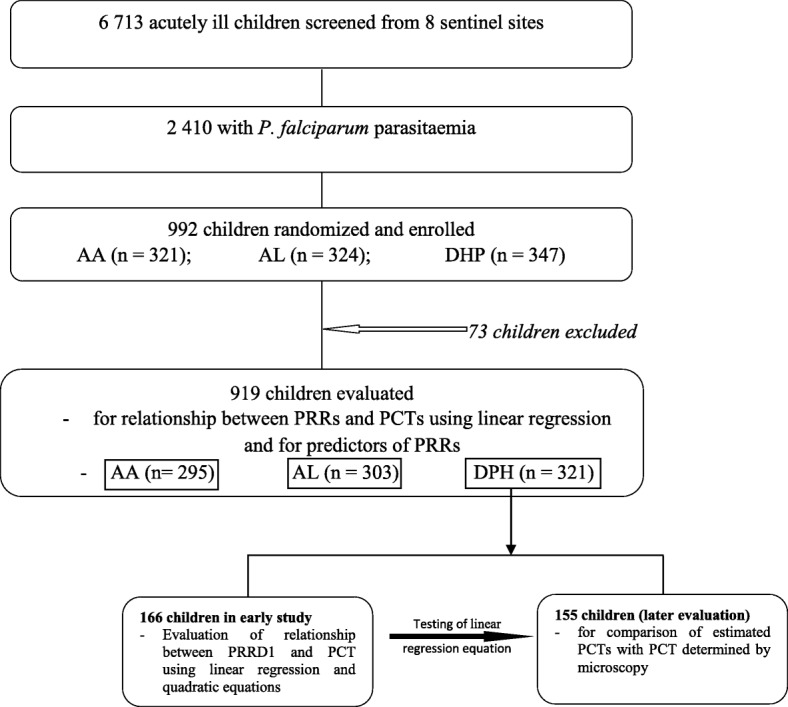
Study profile

Patients allotted to DHP treatment were divided into two halves; that is, an ‘initial or early half’ and the ‘later half’. In the ‘early half’, the relationship between PRRD1 and PCT determined by microscopy was explored using linear regression and quadratic equations. The linear regression equation generated in the ‘early half’ was used to estimate PCT in the remaining ‘later half’ of DHP-treated children. The estimated PCT was then compared with the PCT determined by microscopy in the same patient in a blinded manner. The study profile is shown in Fig. 1.

### Statistical analysis

Assuming a cure rate of 100% for AL and 95% for AA or DHP, respectively, and a 5% dropout rate, we estimated a minimum of 50 patients per treatment arm in each sentinel site would provide 95% power and a 95% confidence interval (*CI*) [8]. Data were analysed using Epi Info™ version 6 software (Centers for Disease Control and Prevention, Atlanta, GA, USA) [15] and the statistical program SPSS for Windows version 20.0 (SPSS Inc., Chicago IL, USA) [16].

Variables considered in the analysis were related to the densities of *P. falciparum* asexual forms, their reduction ratios (PRRD1/PRRD2) and PCT. Proportions were compared by calculating *χ*^2^ using Yates’s correction, Fisher’s exact or Cochran-Mantel-Haenszel tests. Normally distributed, continuous data were compared using the Student’s *t*-test and analysis of variance (ANOVA), followed by Tukey’s range test. Kaplan-Meier estimator and pairwise log-rank tests were used to determine cumulative risk of persistent parasitaemia three days post-treatment initiation. Univariate analysis and stepwise multiple logistic regression models were used to test the association between clinical, parasitological or haematological parameters, and PRRD1 > 5000 per half cycle or PRRD2 ≥ 10 000 per cycle and independent predictors of these parameters, respectively. The relationships between any of the two parameters, that is, PRRD1 and PCT, PRRD2 and PCT, PRRD1 and PRRD2, were assessed by linear regression or quadratic coefficients, and the equations generated, where applicable, were used for estimating PCTs. In all 919 children treated with the three ACTs, agreement between PCTs generated by linear regression equations was assessed using the Bland-Altman analysis [17]. Similarly, in a subset of 166 DHP-treated children, the agreement between PCTs estimated from the linear regression equation of the relationship between PRRD1 and PCT, and PCTs estimated from the quadratic equation of the relationship between PRRD1 and PCT was assessed using the Bland-Altman analysis [17].

## Results

### Patients’ characteristics at presentation

Overall, 992 children (321, 324 and 347 children in AA, AL and DHP treatment groups, respectively) were enrolled in the study. Of these, 73 children (26, 21 and 26 children in the AA, AL and DHP treatment groups, respectively) were withdrawn prematurely during the first week of follow-up. The baseline characteristics of the 919 children who completed at least one week of follow-up are shown in Table 1.

**Table 1:** Baseline characteristics of 919 children enrolled in the study

Variables	AA (*n* = 295)	AL (*n* = 303)	DHP (*n* = 321)	All (*n* = 919)	*P* value
Male:Female	153:142	159:144	183:138	495:424	0.37
Age (month)					
Mean	39	40.5	38.5	39.3	0.3
95% *CI*	37.2–40.9	38.6–42.3	36.6–40.3	38.3–40.4	
No. ≤ 12 months (%)	23 (7.8)	18 (5.9)	29 (9)	70 (7.6)	0.35
Duration of illness (day)					
Mean	4.2	3.8	3.9	4	0.54
95% *CI*	3.7–4.7	3.3–4.3	3.5–4.3	3.7–4.2	
Weight (kg)					
Mean	13.4	13.5	13.3	13.3	0.18
95% *CI*	13–13.9	13–14	13–13.6	13–13.6	
Temperature (°C)					
Mean	37.8	37.9	37.8	37.8	0.44
95% *CI*	37.6–37.9	37.8–38	37.7–37.9	37.7–37.9	
No. with					
≥ 37.5 °C (%)*	182 (61.7)	195 (64.4)	193 (60.1)	570 (62)	0.55
≥ 40 °C (%)	21 (7)	22 (7.3)	28 (8.7)	81 (8.8)	0.37
Haematocrit (%)					
Mean	30.5	30.3	30.9	30.6	0.3
95% *CI*	29.9–31.1	29.7–30.9	30.3–31.5	30.3–30.9	
No with anaemia (%)	113 (38.3)	120 (39.6)	104 (32.4)	337 (36.7)	0.16
Mild (%)	106 (35.9)	110 (36.3)	91 (28.3)	307 (33.4)	0.06
Moderate (%)	6 (2)	10 (3.3)	13 (4)	29 (3.2)	0.35
Severe (%)	1 (0.3)	0 (0)	0 (0)	1 (0.1)	–
Parasitaemia (/μl)					
Geometric mean	15 518	16 254	15 185	15 638	0.87
Range	2000–200 000	2003–200 000	2000–200 000	2000–200 000	
Gametocytaemia (%)	20 (6.8)	9 (3)	19 (5.9)	48 (5.2)	0.09

*Overall, 570 children were febrile at presentation. Geometric mean enrolment parasitaemia was significantly higher in febrile compared to non-febrile children (20 506/μl [range: 2000–200 000; *n* = 570] versus 10 045/μl [2000–198 200; *n* = 349], respectively; *P* < 0.0001)

*AL*: Artemether-lumefantrine; *AA*: Artesunate-amodiaquine; *DHP*: Dihydroartemisinin-piperaquine; *CI*: Confidence interval

The characteristics of children were similar in all treatment groups. Overall, 570 of the 919 children were febrile at presentation. Geometric mean enrolment parasitaemia was significantly higher in febrile compared to non-febrile children (20 506/μl [range: 2000–200 000; *n* = 570] versus 10 045/μl [range: 2000–198 200; *n* = 349], respectively, *P* <  0.0001).

### Therapeutic responses

Overall, 545 of 919 children (59.3%) had residual asexual parasitaemia one day after treatment initiation. The proportion of children with residual parasitaemia on day 1 was significantly higher in the AL-treated cohort compared with the AA- or DHP-treated cohorts (199 of 303 children [65.7%] versus 173 of 295 children [58.6%] versus 173 of 321 children [53.9%], respectively, *P* = 0.01). Overall, on day 2 after treatment initiation, 213 of 919 children (23.2%) had residual asexual parasitaemia. The proportion of children with residual parasitaemia on day 2 was significantly lower in DHP-treated children compared with AL- or AA-treated children (60 of 321 children [18.7%] versus 83 of 303 children [27.4%] versus 70 of 295 children [23.7%], respectively; *P* = 0.04). On day 28, the overall PCR-corrected adequate clinical and parasitological responses (ACPR) was 588 of 596 children (99.1, 95% *CI*: 97.8–100). The PCR-corrected ACPR was 100% in DHP-treated children. The PCR-corrected ACPR on day 28 for AA- and AL- treated children was similar (189 of 193 children [98.1, 95% *CI*: 96.2–100] versus 187 of 191 children [99.2, 95% *CI*: 95.8–100], respectively, *P* = 1.0).

### Relationship between PRRs and PCT

Overall, for all three treatment regimens, the mean PCT was 1.9 days (95% *CI*: 1.8–1.9, range: 1–5). Parasite clearance was significantly faster in children treated with DHP compared to those treated with AA or AL (1.8 days [95% *CI*: 1.7–1.9, range: 1–5, *n* = 321] versus 1.9 days [95% *CI*: 1.8–2, range: 1–4, *n* = 295] versus two days [95% *CI*: 1.9–2.1, range: 1–4, *n* = 303], respectively, *P* = 0.005). In a *post-hoc* comparison, mean PCT was similar in AA- and AL-treated children (*P* = 0.42).

**Fig. 2:**
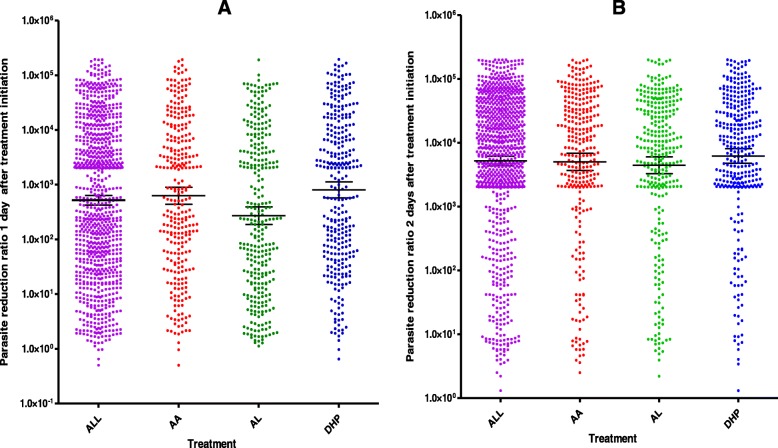
Individual plots of PRR one day (**a**) and two days (**b**) after treatment initiation in all 919 children (purple dots), and those treated with AA (red dots), AL (green dots) or DHP (blue dots). The middle horizontal lines represent geometric means; lines above and below the middle horizontal lines represent 95% *CI*s. AA: Artesunate-amodiaquine; AL: Artemether-lumefantrine; *CI*: Confidence interval; DHP: Dihydroartemisinin-piperaquine

For all three treatment regimens, the overall geometric mean PRRD1 was 518 (95% *CI*: 421–6377, range: 0.5–200 000, *n* = 919). The PRRD1 value was significantly lower in children treated with AL compared with children treated with DHP or AA (270 [95% *CI*: 187–391, range 1.1–191 000, *n* = 303] versus 627 [95% *CI*: 438–898, range: 0.5–191 000, *n* = 321] versus 805 [95% *CI*: 574–1129, range: 0.7–200 000, *n* = 295], respectively, *P* < 0.0001) (see Fig. 2a). In a *post-hoc* comparison, using the Mann-Whitney U test, the geometric mean PRRD1 was significantly lower in children treated with AL compared with those treated with AA (*P* = 0.001) or DHP (*P* < 0.0001), but it was similar in AA- and DHP-treated children (*P* = 0.38).

**Fig. 3:**
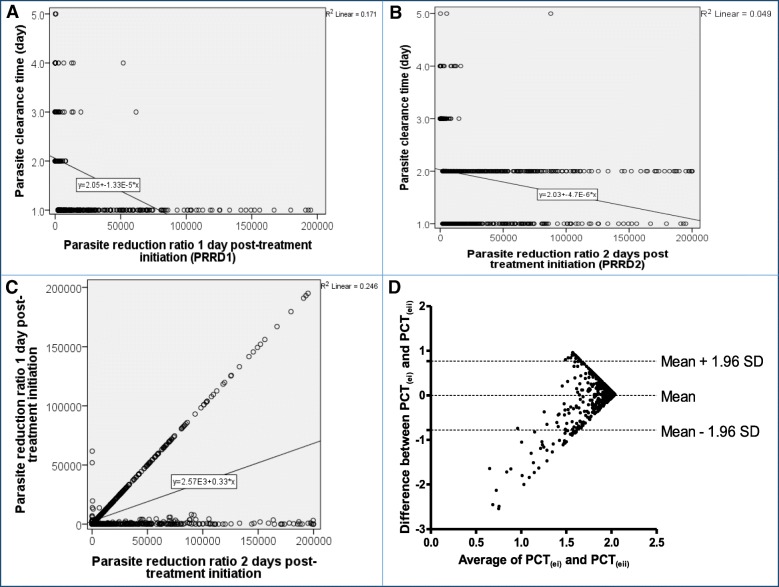
Relationship between PCT and PRR one (**a**) or two (**b**) days after treatment initiation; between PRR one day and two days after treatment initiation (**c**) by linear regression; and Bland-Altman plot of PCTs calculated using linear regression equations of plots A and B (**d**) Note the regression equation in each of plots A, B and C (*P* < 0.0001 for each plot). The *p* value for Bland-Altman plot showed insignificant bias (*P* = 0.7). PCT: Parasite clearance time; PRR: Parasite reduction ratio.

Overall, for all three treatments, there was a significantly negative correlation between PRRD1 and PCT (ρ = 0.76, *P* < 0.0001) (see Fig. 3a). When individual treatments were considered, there was also a significantly negative correlation between PRRD1 and PCT (ρ = 0.78, 0.45 and 0.76; *P* < 0.0001 each for AA, AL and DHP, respectively).

For all three treatment regimens, the overall geometric mean PRRD2 was 5200 (95% *CI*: 4391–6157, range 1.3–200 000, *n* = 919), and it was similar for all three treatments (5034 [95% *CI*: 3691–6864, range: 2.5–199 687] versus 4439 [95% *CI*: 3276–6016, range: 2.2–200 000] versus 6220 [95% *CI*: 4772–8108, range: 1.3–200 000] in AA-, AL- and DHP-treated children, respectively, *P* = 0.51) (see Fig. 2b). Similarly, for all three treatments, there was a significantly negative correlation between PRRD2 and PCT (ρ = 0.47, *P* < 0.0001) (see Fig. 3b). When individual treatments were considered, there was also a significantly negative correlation between PRRD2 and PCT (ρ = 0.48, 0.54 and 0.40; *P* < 0.0001 each for AA, AL and DHP, respectively).

Overall, for all three treatments, PRRD1 correlated significantly positively with PRRD2 (ρ = 0.51, *P* < 0.0001) (see Fig. 3c). The linear regression equations for the correlation analysis between PCT and PRRD1 or PRRD2 were PCT_ei_ = 2.05 – (1.33 × 10^− 5^ × PRRD1) or PCT_eii_ = 2.03 – (4.7 × 10^− 6^ × PRRD2), respectively. From these equations, PCTs estimated from PRRD1 (PCT_ei_) correlated significantly positively with PCTs estimated from PRRD2 (PCT_eii_) (ρ = 0.5, *P* < 0.0001). In a Bland-Altman analysis (see Fig. 3d), the limit of agreement between PCT_ei_ and PCT_eii_ was narrow and the bias was statistically insignificant (limit of agreement = − 0.078–0.077, bias = − 0.005, *P* = 0.7).

### Factors contributing to high PRRs

#### Predictors of PRR > 5 × 10^3^ one day after treatment initiation

Overall, 254 of 919 children (27.6%) had PRRD1 > 5000 per half cycle. Age > 15 months, enrolment parasitaemia > 10 000/μl and treatment with DHP independently predicted PRRD1 > 5000 per half cycle (see Table 2).

**Table 2:** Predictors of parasite reduction ratio > 5000 one day after initiation of artemisinin-based combination therapies in acutely young malarious children

Variable	Total No.	No. with PRRD1 > 5000	*OR* (95% *CI*)	*P* value	a*OR* (95% *CI*)	*P* value
Gender						
Female	424	119	1			
Male	495	135	1.0 (0.7–1.3)	0.85	–	–
Age (month)						
≤ 15	103	19	1		1	
> 15	810	232	1.8 (1.1–3.0)	0.04	2.0 (1.0–4.1)	0.048
Duration of illness (days)						
≤ 2	269	82	1			
> 2	334	121	1.3 (0.9–1.8)	0.16	–	–
Haematocrit at presentation (%)						
< 30	652	170	1		1	
≥ 30	247	83	1.4 (1.0–2.0)	0.03	1.2 (0.8–1.9)	0.3
Haematocrit on day 1 (%)						
≥ 30	483	130	1			
< 30	407	120	1.1 (0.8–1.5)	0.44	–	–
History of fever before treatment						
Absent	170	41	1			
Present	749	213	1.3 (0.9–1.9)	0.3	–	–
Temperature at presentation (°C)						
≤ 37.4	349	88	1			
> 37.4	570	166	1.2 (0.9–1.6)	0.23	–	–
Fever on day 1*						
Absent	797	225	1			
Present	103	26	0.9 (0.5–1.4)	0.6	–	–
Enrolment Parasitaemia						
≤ 10^4^	370	44	1		1	
> 10^4^	349	210	4.6 (3.2–6.6)	< 0.0001	4.8 (3.0–7.5)	< 0.0001
Gametocytaemia						
Absent	871	243	1			
Present	48	11	0.8 (0.4–1.5)	0.56	–	–
Drug treatment						
AL	303	68	1			
AA	295	85	1.4 (1.0–2.0)	0.09	–	–
DHP	321	101	1.6 (1.1–2.3)	0.01	1.8 (1.2–2.6)	0.003

*PRRD1* Parasite reduction ratio 1 day post-treatment initiation, *AL* Artemether-lumefantrine, *AA* Artesunate-amodiaquine, *DHP* Dihydroartemisinin-piperaquine, *CI* Confidence interval, *OR* Odd ratio, a*OR* Adjusted odd ratio

*Fever 1 day post-treatment initiation

#### Predictors of PRR ≥ 10^4^ two days after treatment initiation

Overall, 442 of 919 children (48%) had PRRD2 ≥ 10 000 per cycle. An age > 30 months, haematocrit ≥31% at presentation, enrolment body temperature > 37.4 °C, enrolment parasitaemia > 100 000/μl, PRR > 1000 one day after treatment initiation and an absence of gametocytaemia at presentation were associated with PRRD2 ≥ 10 000 per cycle and independently predicted of PRRD2 ≥ 10 000 per cycle (see Table 3).

**Table 3:** Predictors of parasite reduction ratio ≥ 10 000 two days after initiation of artemisinin-based combination therapies in acutely malarious young children

Variable	Total No.	No. with PRRD2 ≥ 10 000	*OR* (95% *CI*)	*P* value	a*OR* (95% *CI*)	*P* value
Gender						
Female	424	202	1			
Male	495	240	1.0 (0.8–1.3)	0.85	–	–
Age (month)						
≤ 30	284	121	1		1	
> 30	629	320	1.4 (1.1–1.9)	0.02	1.4 (1.0–1.9)	0.03
Duration of illness (days)						
≤ 2	269	163	1			
> 2	334	194	1.3 (0.8–2.0)	0.59	–	–
Haematocrit at presentation (%)						
< 31	465	201	1		1	
≥ 31	434	236	1.6 (1.2–2.0)	0.001	1.5 (1.1–1.9)	0.01
Haematocrit on day 1 (%)						
< 30	407	123	1			
≥ 30	483	226	0.8 (0.6–1.0)	0.11	–	–
History of fever before treatment						
Absent	170	75	1			
Present	749	367	1.2 (0.9–1.7)	0.29	–	–
Temperature at presentation (°C)						
≤ 37.4	349	125	1		1	
> 37.4	570	317	2.2 (1.7–3.0)	< 0.0001	2.0 (1.5–2.7)	< 0.0001
Fever on day 1*						
Absent	797	394	1			
Present	103	47	0.9 (0.6–1.3)	0.53	–	–
Enrolment Parasitaemia						
≤ 10^5^	823	380	1		1	
> 10^5^	96	62	2.1 (1.4–3.3)	< 0.0001	1.9 (1.2–3)	0.01
PRRD1						
≤ 10^3^	501	205	1		1	
> 10^3^	418	237	1.9 (1.5–2.5)	< 0.0001	2.1 (1.6–2.7)	< 0.0001
Gametocytaemia						
Present	48	10	1		1	
Absent	871	432	3.7 (1.8–7.6)	< 0.0001	2.4 (1.1–5.0)	0.02
Drug treatment						
AL	303	141	1			
AA	295	144	1.1 (0.8–1.5)	0.64	–	–
DHP	321	157	1.1 (0.8–1.5)	0.61	–	–

*PRRD1* Parasite reduction ratio 1 day post-treatment initiation, *PRRD2* Parasite reduction ratio 2 days post-treatment initiation, *AL* Artemether-lumefantrine, *AA* Artesunate-amodiaquine, *DHP* Ddihydroartemisinin-piperaquine, *CI* Confidence interval, *OR* Odd ratio, a*OR* Adjusted odd ratio

*Fever 1 day post-treatment initiation

### Asexual parasite positivity on day 3

Asexual parasites were detected in peripheral blood three days after treatment initiation in 19 of 919 children (2%). The cumulative probability of persistent parasitaemia for three days after treatment initiation was significantly higher in AL-treated compared with DHP- or AA-treated children (log-rank statistic = 9.18, *P* = 0.01). PCR amplification was possible pre-treatment (on day 0) in 636 of 842 samples (75.5%). The PCR-corrected APPD3 was 12 of 636 (1.9%). The clinical and parasitological features of the children with delayed clearance of parasitaemia were not significantly different from those without parasitaemia on day 3 (data not shown), making it impossible (due to the very small number of children with APPD3) to evaluate the risk factors for APPD3.

### Evaluation of the relationship between PRRD1 and PCT in DHP-treated children

Characteristics of patients enrolled in the prospective evaluation compared to PCT estimates by linear regression and microscopy.

Because parasite clearance was significantly faster in DHP-treated children and DHP treatment predicted PRRD1 > 5000 per half cycle time, an evaluation of the relationship between PRRD1 and PCT was carried out prospectively in all 321 DHP-treated children. Table 4 shows the characteristics of the 166 children in the early phase of evaluation of the relationship between PRRD1 and PCT and of the 155 children in the later phase of evaluation of PCT estimates from the linear regression equation and the comparison with the PCT determined by microscopy. The characteristics were similar in the two groups. However, gametocyte carriage was significantly higher in children enrolled in the early phase compared with those enrolled in the later phase (15 of 166 children versus 4 of 155 children; *P* = 0.02).

**Table 4:** Baseline characteristics and parasitological responses in dihydroartemisinin-piperaquine-treated children who participated in evaluation of the comparison of estimates of parasite clearance time derived from linear regression equation and microscopy-determined parasite clearance time

Variables	Initial phase (*n* = 166)	Later phase (*n* = 155)	All (*n* = 321)	*P* value
Male: Female	90:76	93:62	183:138	0.35
Age (month)				
Mean (95% *CI*)	37.6 (34.9–40.2)	39.5 (36.9–42)	38.1 (36.4–39.7)	0.31
No. ≤ 12 months (%)	16 (9.6)	13 (8.4)	29 (9)	0.84
Duration of illness (day)				
Mean (95% *CI*)	3.8 (3.2–4.4)	4.1 (3.4–4.7)	3.8 (3.4–4.2)	0.53
Weight (kg)				
Mean (95% *CI*)	12.8 (12.2–13.5)	13.1 (12.5–13.7)	12.9 (12.4–13.3)	0.56
Temperature (°C)				
Mean (95% *CI*)	37.8 (37.6–38)	37.8 (37.5–38)	37.8 (37.7–37.9)	0.8
No. with				
≥ 37.5 °C (%)	94 (56.6)	99 (63.9)	193 (60.1)	0.23
≥ 40 °C (%)	15 (9)	13 (8.4)	28 (8.7)	0.99
Haematocrit (%)				
Mean (95% *CI*)	30.6 (29.7–31.5)	31.3 (30.6–32)	30.8 (30.3–31.4)	0.21
No with anaemia (%)	59 (35.5)	45 (29)	104 (32.4)	0.34
Mild (%)	48 (28.9)	43 (27.7)	91 (28.3)	0.65
Moderate (%)	11 (6.6)	2 (1.3)	13 (4)	0.02
Severe (%)	0 (0)	0 (0)	0 (0)	–
Parasitaemia (/μl)				
Geometric mean (Range)	16 319 (2000–200 000)	14 057 (2003–198 000)	15 185 (2000–200 000)	0.27
Gametocytaemia (%)	15 (9)	4 (2.6)	19 (5.9)	0.02
Parasite positivity				
On day 1	87	83	170	0.93
On day 2	29	31	60	0.66
On day 3	5	2	7	0.45
Parasite clearance time (day)				
Mean (95% *CI*)	1.8 (1.6–1.9)	1.8 (1.7–1.9)	1.8 (1.7–1.9)	0.88
PRRD1				
Geometric mean (95% *CI*)	923 (585–1456)	695 (421–1149)	805 (574–1129)	0.46
PRRD2				
Geometric mean (95% *CI*)	7428 (5298–10 415)	5143 (3407–7765)	6220 (4772–8108)	0.3

*PRRD1* Parasite reduction ratio 1 day after treatment initiation, *PRRD2* Parasite reduction ratio 2 days after treatment initiation, *CI* Confidence interval

### Evaluation of the relationship between PRRD1 and PCT, and generation of regression equations

**Fig. 4:**
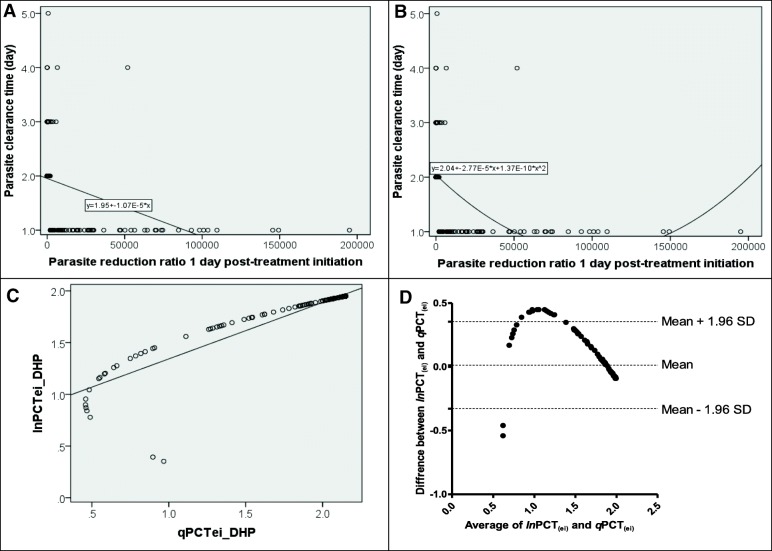
Relationship between PCT and PRRD1 in the 166 children treated with DHP in the initial phase. Panels **a** and **b** between observed PCT and PRRD1 by linear and quadratic equations, respectively, in the initial 166 children; Panel **c**: between estimated PCTs from linear (*ln*PCT_ei_) and quadratic regression equation (*q*PCT_ei_) in the initial 166 children; Panel **d** Bland-Altman plot of PCTs estimated from linear and quadratic regression equations of plots A and B. *P*-values for Bland-Altman plots showed insignificant bias (*P* = 0.89). PCT: Parasite clearance time; PRRD1: Parasite reduction ratio one day post-treatment initiation

In the 166 children enrolled in the early phase, the mean PCT determined by microscopy and the geometric mean PRRD1 were 1.8 days (95% *CI*: 1.6–1.9, *n* = 166) and 922 (range: 0.7–195 000, *n* = 166), respectively. There was a significantly negative correlation between PCT determined by microscopy and PRRD1 (ρ = 0.76, *P* < 0.0001) (see Fig. 4a and b). The linear and quadratic regression equations for the correlation between PCT and PRRD1 were *ln*PCT_ei_ = 1.95 – (1.07 × 10^− 5^ × PRRD1) (see Fig. 4a) and *q*PCT_ei_ = 2.04 – (2.77 × 10^− 5^ × PRRD1) + (1.34 × 10^− 10^ × PRRD1^2^) (see Fig. 4b), respectively. Using the linear and quadratic regression equations, the estimated PCTs (*ln*PCT_ei_ and *q*PCT_ei_) correlated significantly positively in the same patients (ρ = 1.0; *P* < 0.0001) (see Fig. 4c). In the Bland-Altman analysis, the limits of agreement between *ln*PCT_ei_ and *q*PCT_ei_ was narrow and the bias was statistically insignificant (limit of agreement = − 0.3289–0.356, bias = 0.0138, *P* = 0.89) (see Fig. 4d), indicating that both could be used interchangeably in the 166 children enrolled in the early phase of the prospective study.

### Comparison of PCTs using linear regression equation generated in the early phase with the PCTs estimated by microscopy in children enrolled in the later phase (estimated versus observed PCT)

**Fig. 5:**
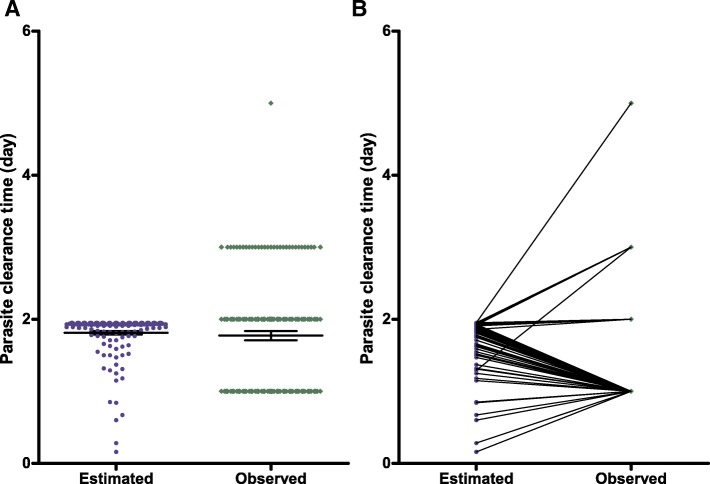
Scatter dot plots (**a**) and aligned dot plots (**b**) of individual PCTs estimated by linear regression equation (blue dots) and those determined by microscopy (green dots) in the same patient in a cohort of 155 DHP-treated children. The linear regression equation used to estimate PCTs was generated from the relationship between observed PCT and PRRD1 in an initial cohort of 166 DHP-treated children and tested in a later cohort of 155 DHP-treated children prospectively. Horizontal bars in (**a**) indicate mean and 95% *CI*. PCT: Parasite clearance time; PRRD1: Parasite reduction ratio one day post-treatment initiation; DHP: Dihydroartemisinin-piperaquine; *CI*: Confidence interval

Since the linear regression equation and quadratic equation could be used interchangeably in Bland-Altman analyses, the simpler linear regression equation was chosen to compare estimates of PCT using this equation with the PCT determined by microscopy in the same patient in the later phase (*n* = 155). Overall, the group mean PCT estimated using a linear regression equation in the 155 prospectively studied children was similar to that determined by microscopy in the same patient (1.81 days [95% *CI*: 1.76–1.86] versus 1.78 days [95% *CI*: 1.65–1.91), respectively, *P* = 0.66) (see Fig. 5a). The paired PCT values in the same patient were also similar compared using the paired t-test (*P* = 0.61) (see Fig. 5b).

## Discussion

We explored the relationship between PRR one or two days after initiation of ACTs and PCTs in young acutely malarious children, and used PRRD1 after treatment initiation as an alternative measure of responsiveness to ACTs. Our study showed agreement between measures of responsiveness determined by both PRRs.

The significantly faster PCT in DHP-treated children compared to other ACTs and the significantly lower PRRD1 in AL-treated children compared to other ACTs were not unexpected and could have been due to a number of factors. These include the less frequent use of DHP compared to AL as evidenced, for example, by the non-inclusion of DHP in the National Guidelines as one of the recommended ACTs in Nigeria [18], and distribution data showing lesser utilisation of AA (approximately 10–25%) both in private and public sectors in the six states of Nigeria targeted for ACT coverage under The Global Fund and World Bank Project. In addition, after seven years of adoption, AA is more efficacious than AL in acutely malarious Nigerian children [8, 13]. It is also likely that the less frequent use of DHP and AA, and the little or no use of chloroquine following adoption of ACTs as first-line antimalarials, partly contributed to the superior efficacy of artemisinin-4-aminoquinoline combinations over AL [8].

In all evaluations of relationships between PRRD1 or PRRD2 and PCT, and between the two PRRs, there were significant correlations between all the parameters evaluated. However, all the values of ρ were < 1 in all evaluations except the relationship between PRRD1 and PCT estimated by linear and quadratic equations (*ln*PCT_ei_ and *q*PCT_ei_), where ρ = 1.0 (see Fig. 3c). The estimates of PCTs generated by linear or quadratic equations for any of the pair’s relationships showed that PCT estimates derived using PRRD1 and PRRD2 can be used interchangeably on Bland-Altman plots. These outcomes were not unexpected.

Of the predictors of PRRD1 or PRRD2, relatively older children and relatively higher values than the cut-offs were common to both measures of responsiveness (see Tables 2 and 3). It is intriguing, considering age as a predictor (Tables 2 and 3), the value of age for predicting PRRD2 was twice that for predicting PRRD1. It is likely the chosen cut-off point of PRRD1 being half of PRRD2 is responsible for the value of ρ = 0.5 in the evaluation of the relationship between PRRD1 and PRRD2. In addition, an evaluation using PRRD1 was done at half the time of the intraerythrocytic cycle of approximately 48 h, the conventional time for the evaluation of PRRD2. Taken together, the finding that the older range in children aged five years have higher PRR values indicates that therapeutic responses across the age band 6–59 months are not uniform even in the setting of full sensitivity of *P. falciparum* to ACTs in endemic areas of Nigeria (see Table 3). A similar finding has been reported in recent studies in other endemic areas of the country [12, 19].

Values of PRRs higher than cut-offs being predictors of the two PRRs confirm full sensitivity of *P. falciparum* to ACTs in these areas, and that ACTs rapidly clear asexual parasitaemias and can prevent their progression to sexual forms [14, 20]. The latter explains the absence of gametocytaemia as a predictor of PRRD2 ≥ 10 000 per cycle. That PRRD1 > 1000 per half cycle is an independent predictor of PRRD2 ≥ 10 000 per cycle confirms the close relationship, interchangeability and the usefulness of PRRD1 as an early measure of responsiveness to ACTs. That fever is a predictor of PRRD2 ≥ 10 000 per cycle is explicable in the context of children with fever at presentation having significantly higher geometric mean parasitaemia than those without fever (see Table 1) and PRRD2 ≥ 10 000 per cycle itself being a predictor of PRRD2 > 10 000 per cycle. An alternative explanation for fever being a predictor of PRRD2 > 10 000 per cycle is that fever, a non-specific host-defence mechanism, (i) may directly enhance the activity of artemisinin derivatives, or (ii) increase clearance of infected erythrocytes by the spleen, or (iii) enhance other mechanisms of parasite clearance following initiation of ACTs. The role of fever at presentation as a predictor of PRRD2 in excess of 10 000 per cycle requires further exploration in future studies. We have no available explanation for enrolment haematocrit > 31% being a predictor of PRRD2 ≥ 10 000 per cycle.

APPD3 > 3% in patients with pre-treatment asexual parasitaemia < 100 000/μl or > 10% of patients with detectable parasitaemia 72 h after initiation of direct observed therapy is used as one of the in vivo measures of reduced artemisinin susceptibility [3, 21]. In the current study, using the PCR-corrected APPD3 value of 1.9% showed there is no evidence of in vivo reduced susceptibility to artemisinin components of ACTs in all endemic areas of Nigeria [8]. The very low value of PCR-corrected APPD3 did not permit the evaluation of risk factors for APPD3 in the present cohort of children treated with ACTs. In areas of low transmission, for example in Southeast Asia, mutations in *P. falciparum* Kelch-13 genes (*Pf*K13 genes) have been associated with APPD3, long half-life of parasitaemia (half-life of five hours or more) and resistance to artemisinin on a ring stage survival assay [22–25]. In Africa, declining responsiveness in *P. falciparum* to ACTs, measured as reducing PRRD1 values between 2005 and 2008, has been reported on the Kenyan coast [26], but there is currently no concrete evidence of artemisinin resistance using *Pf*K13 polymorphisms [25] or half-life of parasitaemia (half-life of 1.2 h or less) in malarious Nigerian children [8, 13, 27].

In falciparum malaria, parasite killing and clearance are first order processes that can be influenced by host, parasite, drug and other factors [5, 14]. The regression equations of the relationship between the PRRs and PCTs in the cohort of children evaluated represent a summation of these factors in a rather generalised manner in endemic areas of Nigeria where there is full sensitivity in *P. falciparum* to ACTs. The relationships between the parameters evaluated will undoubtedly be affected by changes in host, parasite and other factors, if and when resistance develops to artemisinins in these areas. Therefore, there is a need to constantly evaluate the relationship between PRRD1 and PCT in order to detect early changes in the relationship that may indicate declining responsiveness in *P. falciparum* to ACTs, and to put into place appropriate mechanisms for molecular surveillance of mutation in *Pf*K13 genes associated with artemisinin resistance.

Parasite multiplication and declines after antimalarial treatments of sensitive infections are often geometric in nature [5]. Asexual parasite density cut-off for predictors of PRRD1, in the current study, was not the geometric value of the cut-off for predictors of PRRD2. Cut-off for predictors of PRRD1 was proportional, and was based on the fact that PRRD1 was evaluated one day post-treatment initiation as compared to PRRD2 that was evaluated two days post-treatment initiation. In many endemic areas, there are challenges with patient follow-up. Incomplete data collection and data loss may affect study outcomes. The close relationship between PRRD1 and PRRD2, and the finding that PRRD1 > 1000 per half cycle predicts PRRD2 ≥ 10 000 per cycle permit the use of PRRD1 as a measure of not only responsiveness in therapeutic trials but also in estimating PCTs particularly in ongoing clinical studies, providing the relationship between PRRD1 and PCT has been evaluated in the early phase of the ongoing clinical studies.

The limitation of the current study is its use of simple mathematical approaches for evaluating the relationship between PRRs and PCTs, and not factoring other parameters into the analysis, for example, age, parasite staging or parasitaemia half-time. Therefore, there are urgent needs to explore the relationship between PRRD1 and PCT using other mathematical approaches, and impetus to carry out pharmacodynamic-pharmacokinetic modelling of the relationship between PRRD1 and PCT in large populations in areas of non-artemisinin and artemisinin-resistant falciparum malaria. In addition, there is a need for a mathematical model that would predict resistance to ACTs in areas where ACTs are still largely efficacious, for example in Africa, using the relationship between PRRD1 and PCT, or the relationship between PRRD1 and parasitaemia elimination half-time in excess of three hours.

## Conclusions

Parasite reduction ratio 1 day post-treatment initiation (PRRD1) is an early measure (with 24 h) of responsiveness to ACT. It correlates significantly with PRRD2 and PCT (conventional measures of responsiveness to antimalarial chemotherapy). Thus, results and discussion of this study showed estimates of PCT using PRRD1 linear regression equation can be used in ongoing therapeutic efficacy studies to evaluate responsiveness to antimalarial drugs.

## Additional file

Additional file 1: Multilingual abstracts in the five official working languages of the United Nations. (PDF 340 kb)

## Abbreviations

AA: Artesunate-amodiaquine
ACPR: Adequate clinical and parasitological responses
ACT: Artemisinin-based combination therapy
AL: Artemether-lumefantrine
APPD3: Asexual parasite positivity three days post-treatment initiation
*CI*: Confidence interval
DHP: Dihydroartemisinin-piperaquine
*ln*PCT_ei_: Estimated parasite clearance time using linear regression equation
PCR: Polymerase chain reaction
PCT: Parasite clearance time
PRR: Parasite reduction ratio
PRRD1: Parasite reduction ratio one day post-treatment initiation
PRRD2: Parasite reduction ratio two days post-treatment initiation
*q*PCT_ei_: Estimated parasite clearance time using quadratic equation
