# Supplementary material for: Parasite reduction ratio one day after initiation of artemisinin-based combination therapies and its relationship with parasite clearance time in acutely malarious children
Source: Infect Dis Poverty. 2018 Dec 7;7:122. doi: 10.1186/s40249-018-0503-7 (PMC6284283; doi:10.1186/s40249-018-0503-7)
Supplement: Supplementary file 1 — Multilingual abstracts in the five official working languages of the United Nations. (PDF 340 kb) [file 40249_2018_503_MOESM1_ESM.pdf]

Translation of the abstract into the five official working languages of the United Nations

تقليل نسبة الطفيليات بعد يوم واحد من البدء باستخدام علاجات تركيبها الأساسي من مادة الأرتيميسنين وعلاقتها بوقت التطهير الطفيلي للأطفال المصابين بالفعل بالملاريا .

كاظم أنكو ، و جودنوين ناتدامو، و شيمير أجومو، و كريستيان تي حابي ، و اونيكيب ايه فولارين ، و جريس اوه جوبوتشو ، و اولجابينجا موكولو ، وفينومو فينومو ، و جوي سي ايبنيب ، و نما جيا ، و جوس امبي ، و روبينسون واماندا ، و جورج امبيتشيب ، و اولووابنمي كيه باسورون ، و اولوبنمي ايه ويوي ، و سيكيرو آمو ، و ننينا ايزيجوي ، و ستيفين اوتش ، و بايو فاتونمبي ، و اكينتوند سونمي .

نبذة مختصرة

خلفية الموضوع في الأنيميا المنجلية الحادة تقليل نسبة الطفيل اللاجنسي بعد يومين من بدء العلاج . واستخدمت (PRRD2) - نسبة الحد من الطفيل اللاجنسي من اليوم صفر حتى اليوم الثاني- كطريقة لقياس سرعة إزالة الطفيليات ومدى فعالية مشتقات الأرتيميسينين . ومع ذلك هناك القليل من التقييم لتدابير بديلة ، على سبيل المثال : طريقة تقليل نسبة الطفيل بعد يوم واحد من بدء العلاج (PRRD1) وعلاقتها بفترة التطهير الطفيلي (PCT) أو PRRD2 . قيمت هذه الدراسة استخدام (PRRD1) كمقياس الاستجابة للأدوية المضادة للملاريا .

الوسائل: تم علاج الأطفال الذين يعانون إصابة حادة بالملاريا بـ [ارتيسنات امودياكاين (AA) و ارتيميثر لومفانترين (AL) أو داي هيدروارتيميسنين بيبراكوين (DHP)، العلاقات بين طريقة تقليل نسبة الطفيل بعد يوم واحد من بدء العلاج PRRD1 أو تقليل نسبة الطفيل بعد بدء العلاج من اليوم صفر حتى اليوم الثاني PRRD2 وفترة التطهير الطفيلي PCT ، وبين PRRD1 و PRRD2 تم تقييمها باستخدام الانحدار الخطي . تم تقييم التوافق بين تقديرات فترة التطهير الطفيلي باستخدام معادلات الانحدار الخطي لــ PRRD1 و PRRD2 من خلال استخدام تحليل بلاند ألتمان .

تم تقييم توقعات لــ PRRD1 > 5000 لكل نصف دورة و PRRD2 ≥ 10000 لكل دورة باستخدام تدرج نماذج الانحدار اللوجيستي المتعدد . باستخدام معادلة الانحدار الخطي للعلاقة بين PRRD1 و PCT والتي تولدت سابقا في نصف عدد الأطفال المعالجين بالــ DHP خلال مرحلة الدراسة المبكرة . فقد تمت المقارنة بين تقديرات PCT بطريقة مستقبلية عمياء و تحديد PCTs من خلال الفحص المجهري أثناء مرحلة الدراسة اللاحقة على النصف المتبقي من الأطفال .

النتائج: في 919 طفل مصاب بالملاريا كان PRRD1 أعلى بكثير في حــالة العلاج بـ DHP و AA بالمقارنة ب AL الذي تم علاج الأطفال بـــه (P < 0.0001). ترتبط قيم PRRD 1 و PRRD 2 بشكل ايجابي كبير مع بعضها البعض (0.0001>) ، وترتبط بشكل سلبي كبير مع قيم PCT لكل 0.0001> . تقديرات الــ PCT من معادلات الانحدار الخطي لــ PRRD1 و PRRD2 تظهر تحيز ضعيف على الرسم البياني لبلاند ألتمان (0.7 =) مشيرة إلى أن هذه التقديرات يمكن استخدامها بالتبادل . في العرض التقديمي، عمر> 15 شهرًا، ونسبة طفيل أكثر من 10000 / ملم من الدم تنبأ علاج الــ DHP بشكل مستقل ،وجود PRRD1 أكثر من 5000 لكل نصف دورة ، بينما في الأعمار التي تزيد على 30 شهرا فإن الهيماتوكريت (نسبة كرات الدم الحمراء بالدم) بنسبة تساوي أو تزيد على 31 % مع حرارة جسم أعلى من 37.4 درجة مئوية ، تكون نسبة الطفيل > 10000 /ملم -1 وتكون قيمة الــ PRRD1 أكثر من 1000 ولا تنبؤ لعرسية الدم بشكل مستقل ل PRRD2 اكثر من أو تساوي 10000 لكل دورة . باستخدام معادلة الانحدار الخطي التي تولدت خلال المرحلة المبكرة لدى 166 طفلاً تم علاجهم بالــ DHP، تقديرات الــ PCT وال PCT التي تم تحديدها عن طريق الفحص المجهري في ال 155 طفلًا في المرحلة اللاحقة مماثلة في نفس المرضى .

الخلاصةPRRD1 وتقديرات الــ PCT واستخدام معادلة الانحدار الخطي لــ PRRD1 و PCT يمكن الاستفادة منهم في دراسات الفاعلية العلاجية .

Translated from English version into Arabic by Heba Telhady and Maha Husaini, through

TRANSLATORS
WITHOUT BORDERS

急性疟疾患儿采用青蒿素为基础的联合疗法一天后寄生虫减少率及其与寄生虫清除时间的相关性研究

Kazeem Akano, Godwin Ntadom, Chimere Agomo, Christian T. Happi, Onikepe A. Folarin, Grace O. Gbotosho, Olugbenga Mokuolu, Finomo Finomo, Joy C. Ebenebe, Nma Jiya, Jose Ambe, Robinson Wammanda, George Emechebe, Oluwabunmi K. Basorun, Olubunmi A. Wewe, Sikiru Amoo, Nnenna Ezeigwe, Stephen Oguche, Bayo Fatunmbi and Akintunde Sowunmi

摘要

引言：在急性恶性疟疾中，治疗两天后无性原虫减少率(PRRD2) ≥ 10 000 已经作为评估快速清除寄生虫血症和青蒿素衍生物是否有效的标准。然而，目前尚缺乏对替代标准的评估；例如，治疗起始日原虫减少率(PRRD1)及其与寄生虫清除时间(PCT)或 PRRD2 的关系。本研究旨在评估能否使用 PRRD1 作为抗疟药物反应性的评价标准。

方法：使用青蒿琥酯-阿莫地喹(AA)、青蒿醚-卢梅凡汀(AL)或双氢青蒿素-哌喹(DHP)治疗急性疟疾患儿，采用线性回归评价 PRRD1 或 PRRD2 与 PCT、PRRD1 与 PRRD2 之间的关系。用 Bland-Altman 分析评估使用 PRRD1 与 PRRD2 的 PCT 估计值线性回归方程的一致性。运用逐步多元 logistic 回归模型对 PRRD1 > 5000 每半循环和 PRRD2> 10 000 每循环的预测因子进行评价。使用早期研究阶段在一半 DHP 治疗的患儿中产生的 PRRD1 和 PCT 关系的线性回归方程，将 PCT 估计值以前瞻性盲法与后期研究阶段通过显微镜确定的 PCTs 进行比较。

结果：在919例疟疾患儿中，DHP和AA治疗的PRRD1明显高于AL治疗的患儿($P < 0.0001$)。PRRD1 或 PRRD2 值与 PCT 值呈显著负相关(各值 $P < 0.0001$)，彼此间呈显著正相关($P < 0.0001$)。PRRD1 和 PRRD2 的线性回归方程的 PCT 估计值在 Bland-Altman 图($P = 0.7$)上显示偏倚不显著，表明这些估值可以替换使用。年龄 > 15 个月，原虫血症 > 10 000 /μl 血液和 DHP 治疗的独立预测 PRRD1 > 5000 每半循环；年龄 > 30 个月，血细胞压积 ≥ 31%，体温> 37.4 ºC，原虫血症 > 100 000/μl，PRRD1 值 > 1000，且没有 gametocytaemia 可独立预测 PRRD2 ≥ 10000 每循环。使用早期研究 166 名 DPH 治疗患儿生成的线性回归方程，在后期 155 名儿童中，通过显微镜测定的 PCT 估计值和 PCTs 在同一患者中接近。

结论：PRRD1 和使用 PRRD1 与 PCT 线性回归方程来估算 PCT 可用于疗效研究。

Translated from English version into Chinese by Xin-Yu Feng, edited by Jin Chen

CHINESE CENTER FOR DISEASE CONTROL AND PREVENTION
NATIONAL INSTITUTE OF PARASITIC DISEASES

Ratio de réduction du parasite un jour après la mise en place de polythérapies à base d'artémisinine et lien avec le délai d'élimination du parasite chez des enfants atteints de paludisme aigu

Kazeem Akano, Godwin Ntadom, Chimere Agomo, Christian T. Happi, Onikepe A. Folarin, Grace O. Gbotosho, Olugbenga Mokuolu, Finomo Finomo, Joy C. Ebenebe, Nma Jiya, Jose Ambe,

Robinson Wammanda, George Emechebe, Oluwabunmi K. Basorun, Olubunmi A. Wewe, Sikiru Amoo, Nnenna Ezeigwe, Stephen Oguche, Bayo Fatunmbi et Akintunde Sowunmi

Résumé

Contexte : Dans le paludisme à *P. falciparum* aigu, un ratio de réduction du parasite asexué deux jours après le début du traitement (PRRD2) ≥ 10 000 par cycle a été utilisé comme indicateur de l'élimination rapide de la parasitémie et de l'efficacité de l'artémisinine et de ses dérivés. Les autres indicateurs possibles ont cependant été peu évalués, par exemple le taux de réduction du parasite un jour après le début du traitement (PRRD1) et sa corrélation avec le délai d'élimination du parasite (PCT) ou avec le PRRD2. Cette étude évalue l'utilisation du taux de réduction du parasite un jour après le début du traitement (PRRD1) comme indicateur de réponse aux médicaments antipaludéens.

Méthodes : Chez les enfants atteints de paludisme aigu sous traitement combiné par artésunate-amodiaquine (AA), artéméther-luméfantrine (AL) ou dihydroartémisinine-pipéraquine (DHP), les corrélations entre PRRD1 ou PRRD2 et PCT, d'une part, et entre PRRD1 and PRRD2, d'autre part, ont été analysées par régression linéaire. La concordance entre les estimations de PCT utilisant les équations de régression linéaire de PPRD1 et de PRRD2 a été évaluée à l'aide de l'analyse de Bland-Altman. Les indicateurs prédictifs de PRRD1 > 5000 par demi-cycle et PRRD2 ≥ 10 000 par cycle ont été évalués à l'aide de modèles de régression logistique multiniveau. En utilisant l'équation de la régression linéaire de la relation entre PPRD1 et PCT précédemment générée pour la moitié des enfants traités avec la dihydroartémisinine-pipéraquine (DHP) pendant la première phase de l'étude, nous avons comparé prospectivement les estimations de PCT avec les PCT déterminés par la microscopie chez l'autre moitié des enfants au cours de l'étape suivante de l'étude.

Résultats : Chez 919 enfants atteints de paludisme, le ratio PRRD1 était significativement plus élevé chez les enfants traités par DHP et AA que chez ceux traités par la combinaison AL ($P < 0{,}0001$). Les valeurs de PRRD1 ou PRRD2 présentaient une corrélation négative significative avec les valeurs de PCT ($P < 0{,}0001$ dans les deux cas) et une corrélation positive significative entre elles ($P < 0{,}0001$). Les estimations du PCT obtenues par les équations de régression linéaire pour PRRD1 et PRRD2 ont montré un biais négligeable dans le graphique de Bland-Altman ($P = 0{,}7$), ce qui montre que l'une ou l'autre estimation peut être utilisée indifféremment. Lors de la présentation des enfants, les facteurs d'âge > 15 mois, parasitémie > 10 000/µl de sang et traitement avec la combinaison dihydroartémisinine-pipéraquine (DHP) étaient, indépendamment, prédictifs de PRRD1 > 5000 par demi-cycle, tandis que les facteurs d'âge > 30 mois, hématocrite ≥ 31 %, température corporelle > 37,4 °C, parasitémie > 100 000/µl, PRRD1 > 1000 et absence de gamétocytémie étaient indépendamment prédictifs de PRRD2 ≥ 10 000 par cycle. En utilisant l'équation de régression linéaire générée pendant la première phase chez 166 enfants traités avec la dihydroartémisinine-pipéraquine (DHP), les estimations du temps d'élimination du parasite (PCT) et les PCT déterminés au microscope chez 155 enfants dans la phase suivante étaient similaires chez les mêmes patients.

Conclusions : Les estimations de PRRD1 et les estimations du PCT utilisant l'équation de régression linéaire de PRRD1 et de la relation entre PPRD1 et PCT peuvent être utilisées dans les études d'efficacité thérapeutique.

Translated from English version into French by Vero Marie and Eve Anderson, through

Коэффициент уменьшения паразита на следующий день после начала комбинированной терапии на базе артемизинина и его взаимосвязь со временем очищения организма от паразита у детей с острой малярией

Казим Акано, Годвин Нтадом, Шимер Агомо, Кристиан Т. Хаппи, Оникепе А. Фоларин, Грейс О. Гботошо, Олугбенга Мокуоло, Финомо Финомо, Джой К. Ебенебе, Нма Джия, Хосе Амбе, Робинсон Вамманда, Джордж Емечебе, Олувабунми К. Басорун, Олубунми А. Веве, Сикиру Амоо, Нненна Езеигве, Стивен Огуче, Байо Фатунмби и Акинтуде Совунми

Аннотация

История вопроса: При острой тропической малярии в качестве меры быстрого очищения от паразитемии и меры эффективности производных артемизинина использовался коэффициент уменьшения бесполых паразитов два дня после начала лечения (PRRD2) $\geq$ 10 000 за цикл. Однако практически отсутствует оценка альтернативных мер, таких как, например, коэффициент уменьшения паразита через день после начала лечения (PRRD1) и его взаимосвязь со временем очищения организма от паразитов (ВОП) или PRRD2. В данном исследовании оценивается использование PRRD1 как меры восприимчивости к противомалярийым лекарствам.

Методы: У детей с острой малярией, которые лечились артезунат-амодиахином (АА), артеметер-люмефантрином (АЛ) или дигидроартемизинин-пиперахином (ДГП), взаимосвязь между PRRD1 или PRRD2 и PCT, а также между PRRD1 и PRRD2 оценивались с использованием линейной регрессии. Согласование оценок ВОП с использованием уравнений линейной регрессии PRRD1 и PRRD2 было произведено с использованием анализа Бланда-Альтмана. Прогностические показатели PRRD1> 5000 за полцикла и PRRD2 $\geq$ 10 000 за цикл оценивались с использованием моделей ступенчатой множественной логистической регрессии. Используя линейное регрессионное уравнение взаимосвязи между PRRD1 и PCT, предварительно полученными у половины детей, проходивших лечение ДГП на ранней стадии исследования, было произведено сравнение оценок ВОП перспективным слепым способом с ВОП, полученными с помощью микроскопии на более поздней стадии исследования у оставшейся половины детей.

Результаты: Среди 919 детей, страдающих малярией, PRRD1 был значительно выше у тех, которые прошли лечение ДГП и АА по сравнению с теми, которых лечили АЛ ($P < 0.0001$). Значения PRRD1 или PRRD2 продемонстрировали высокую степень негативной корреляции со значениями ВОП ($P < 0.0001$ по каждому) и высокую степень кореляции друг с другом ($P < 0.0001$). Оценки ВОП, полученные с помощью уравнений линейной регрессии по PRRD1 и PRRD2, показали несущественное отклонение на графике Блэнда-Альтмана ($P = 0.7$), что указывает на взаимозаменяемость при использовании оценочных показателей. При постановке диагноза возраст > 15 месяцев, паразетимия > 10000/µl крови и лечение ДГП

независимо друг от друга прогнозировали PRRD1 > 5000 за полцикла, в то время как возраст > 30 лет, гематокритное число ≥ 31%, температура тела > 37.4 ^{o}C, паразитемия > 100000/μl^{-1}, значение PRRD1 > 1000 и отсутсвие гаметоцитов в крови независимо друг от друга прогнозировали PRRD2 ≥ 10 000 за цикл. Используя уравнение линейной регрессии, полученное на ранней стадии среди 166 детей, которые лечились ДГП — оценки ВОП и ВОП, определённые с помощью микроскопии у 155 детей на более поздней стадии, были аналогичны по тем же пациентам.
Выводы: PRRD1 и оценки ВОП с применением линейного регрессионного уравнения переменных PRRD1 и ВОП могут быть использованы в исследованиях терапевтической эффективности.

Translated from English version into Russian by Ira Kulinevych and Liudmila Tomanek, through

TRANSLATORS
WITHOUT BORDERS

Tasa de disminución del parásito luego del inicio de la combinación de terapias basadas en artemisinina y su relación con el tiempo de eliminación del parásito en niños sumamente palúdicos.

Kazeem Akano, Godwin Ntadom, Chimere Agomo, Christian T. Happi, Onikepe A. Folarin, Grace O. Gbotosho, Olugbenga Mokuolu, Finomo Finomo, Joy C. Ebenebe, Nma Jiya, Jose Ambe, Robinson Wammanda, George Emechebe, Oluwabunmi K. Basorun, Olubunmi A. Wewe, Sikiru Amoo, Nnenna Ezeigwe, Stephen Oguche, Bayo Fatunmbi y Akintunde Sowunmi

Resumen

Contexto: En paludismo falciparum grave, la disminución de la tasa del parásito asexual dos días luego del inicio del tratamiento (PRRD2) > 10 000 cada ciclo ha sido utilizado como medida para la rápida eliminación de la parasitemia y eficacia de derivados de artemisinina. Sin embargo, hay pocos estudios sobre medidas alternativas; por ejemplo, la disminución un día después del tratamiento (PRRD1) y su relación con el tiempo de eliminación del parásito (PCT por sus siglas en inglés) o PRRD2. Este estudio evaluó el uso del PRRD1 como una medida de respuesta a drogas antimalarias.
Métodos: Se evaluaron las relaciones entre PRRD1 o PRRD2 y PCT, y entre PRRD1 y PRRD2 con el uso de la regresión lineal en niños sumamente palúdicos tratados con artesunado-amodiaquina (AA), artemetero-lumefantrina (AL) o dihidroartemisinina-piperaquina (DHP). Se evaluó el acuerdo entre las estimaciones del PCT con el uso de las ecuaciones de regresión lineal PRRD1 y PRRD2 utilizando el análisis Bland-Altman. Se evaluaron indicadores de PRRD1 > 5000 cada medio ciclo y PRRD2 > 10 000 cada ciclo con el empleo de modelos de regresión de logística múltiple. Se compararon las estimaciones PCT de una manera anticipada con las PCT determinadas por microscopio durante la última fase del estudio en la otra mitad utilizando la ecuación de

regresión lineal de la relación entre PRRD1 y PCT generada de manera previa en la mitad de los niños tratados con DHP durante la primera fase del estudio.

Resultados: En 919 niños diagnosticados con paludismo, el PRRD1 resultó ser significativamente más alto en aquellos tratados con DHP y AA en comparación con niños tratados con AL (*P* < 0,0001). Los valores de PRRD1 o PRRD2 tuvieron una correlación significativamente negativa con los valores PCT (*P* < 0,0001 por cada uno) y significativamente positiva entre sí (*P* < 0,0001). Las estimaciones del PCT a partir de las ecuaciones de regresión lineal para PRRD1 y PRRD2 mostraron una insignificante parcialidad en el plan Bland-Altman (*P* = 0,7), lo que indica que las estimaciones pueden utilizarse de manera intercambiable. Hasta la fecha, edad > 15 meses, parasitemia > 10 000/µl de sangre y tratamiento DHP independientemente predecido PRRD1 > 5000 cada medio ciclo, mientras que la edad > 30 meses, hematocritos > 31 %, temperatura corporal > 37,4 ^{o}C, parasitemia > 10 0000/μl^{-1}, el valor PRRD1 > 1000 y sin gametocitemia independientemente predecida PRRD2 > 10 000 cada ciclo. Al usar la ecuación de regresión lineal generada en una de las primeras etapas en 166 niños tratados con DHP, se observó que las estimaciones de PCT y las de PCT determinadas con un microscopio en los 155 niños de la última etapa fueron similares en los mismos pacientes.

Conclusiones: el PRRD1 y las estimaciones de PCT utilizando la ecuación de regresión lineal de PRRD1 y PCT pueden ser empleados en estudios eficaces terapéuticos.

Translated from English version into Spanish by Agustina Salguero and Rocío Tempone, through

TRANSLATORS
WITHOUT BORDERS
